# Sustainable Epoxidized Guayule Natural Rubber, Blends and Composites with Improved Oil Resistance and Greater Stiffness

**DOI:** 10.3390/ma15113946

**Published:** 2022-06-01

**Authors:** Xianjie Ren, Cindy S. Barrera, Janice L. Tardiff, Katrina Cornish

**Affiliations:** 1 Department of Food, Agricultural and Biological Engineering, Ohio Agricultural Research and Development Center, The Ohio State University, 1680 Madison Avenue, Wooster, OH 44691, USA; ren.447@osu.edu; 2 Research and Advanced Engineering, Ford Motor Company, 2101 Village Rd, Dearborn, MI 48124, USA; cbarre61@ford.com (C.S.B.); jtardiff@ford.com (J.L.T.); 3 Department of Horticulture and Crop Science, Ohio Agricultural Research and Development Center, The Ohio State University, Williams Hall, 1680 Madison Avenue, Wooster, OH 44691, USA

**Keywords:** natural rubber, epoxidation, dynamic mechanical properties, damping performance, oil resistance

## Abstract

Production of petroleum-based synthetic rubbers (SRs) causes an enormous carbon footprint for the rubber industry. Carbon footprint would be reduced if natural rubber (NR) could take a larger market share and replace significant quantities of SR. However, some SRs have higher oil resistance than NRs, and, in applications where these properties are needed, chemically modified NR will be required. Epoxidation is a chemical modification of NR which partially converts unsaturated bonds on the backbone of NR to epoxy groups. In this research, epoxidized guayule natural rubber (EGNR)/guayule natural rubber (GNR) blends and GNR were used to make carbon black (CB) filled composites. The processability, mechanical properties, swelling behaviors and dynamic mechanical properties were characterized at various epoxide fractions. Composites made with EGNR/GNR had higher oil resistance, wet traction and stiffness than GNR composites, although tensile strength and elongation at break were reduced by epoxidation. EGNR is expected to lead to the development of new NR products with similar properties to SR, eroding SR markets and increasing the sustainability of the rubber industry.

## 1. Introduction

Natural rubber (NR) has features like strain-induced crystallization (SIC) and low glass transition temperature, not seen or matched by synthetic rubbers (SRs), but SR still has a larger market share than NR [1]. Although SR production is unsustainable since it is produced from fossil hydrocarbon feedstocks, well-developed polymerization and processing methods makes it popular for various applications. As concerns about the rising carbon footprint of the SR industry increase, approaches to replace SR with more sustainable alternatives are needed. The demand for NR and SR continues to increase, and the NR:SR ratio also has increased in recent years. In 2000, worldwide NR and SR production levels were 6.8 and 10.8 million metric tons, respectively, a ratio of 0.63. In 2017, 13.6 million metric tons of NR and 15.0 million metric tons of SR were produced, a ratio of 0.91 [1]. However, further replacement of SR with NR requires NR to have much better chemical resistance than it naturally has.

Since NR is a nonpolar rubber, the chemical resistance can be improved by increasing its polarity by epoxidation. The NR industry started to produce epoxidized natural rubber in the 1980s [2,3]. NR epoxidation can enhance oil resistance while largely maintaining the superior static and dynamic mechanical properties of NR; epoxidized NR composites had similar oil resistance as polar rubbers, such as nitrile butadiene rubber [4,5]. Epoxidized NR also has higher modulus and wet traction than NR [2,4], as well as lower oxygen and nitrogen permeability than NR, which is desired for membrane gas-separation application [6].

Guayule natural rubber (GNR) is an alternative NR, being developed to increase NR supply security, and could eventually replace HNR and SBR [7]. Epoxidized HNR is well understood and has been commercialized [2,4,8], but epoxidized GNR has been little investigated [9]. In this research, GNR was epoxidized in latex; this solvent-free process has a lower cost of solvent and organic volatile content compared to other epoxidation processes that need organic solvent to precipitate and dissolve GNR and SR [9,10]. Carbon black filled EGNR/GNR blends at different epoxide fractions and GNR composites were manufactured. Energy consumption of compounding, mechanical properties, oil resistance and crosslinking structures were characterized.

## 2. Materials and Methods

GNR was dried from GNR latex, which was produced as described in [11]. The GNR latex was dried in trays at 50 °C for 120 h (HVC 70 series oven, Conceptronic Inc., Portsmouth, NH, USA). Carbon black N330, antioxidant (N-(1,3-dimethylbutyl)-N’-phenyl-p-phenylenediamine (6PPD)) and sulfur-based curing packages (sulfur, stearic acid, zinc oxide and butyl benzothiazole sulfonamide (TBBS)) were generously provided by HB chemicals (Twinsburg, OH, USA). Formic acid (88% concentration by weight) and hydrogen dioxide (30% concentration by weight) were used as epoxidation agents. Non-ionic surfactant, Triton X-100, was purchased from Fisher Scientific (Hampton, NH, USA). Naphthenic oil (NO) (Corsol 2400, R.E. Carroll, Inc., Ewing, NJ, USA) was generously provided by Ford Motor Company (Dearborn, MI, USA).

An amount of 100 g of GNR latex was stabilized with 5 g Triton X-100 and diluted in 150 cm^3^ distilled water at 60 °C for 30 min. GNR latex and surfactant were continuously gently mixed by magnetic stir bar. The concentration of formic acid was diluted from 88% to 30% and used to neutralize GNR latex (pH = 7). To epoxidize GNR latex, formic acid and hydrogen peroxide were added and mixed with stabilized GNR latex for 24 h at 60 °C. The mole ratio of formic acid to hydrogen peroxide was 1:1, while the mole ratios of the polyisoprene unit in GNR latex to formic acid were 1:0.15, 1:0.3 and 1:0.45. The precipitated epoxidized GNR, which formed during the reaction, was washed extensively with tap water. Then, the epoxidized GNR was dried in trays at 50 °C for 72 h (HVC 70 series oven, Conceptronic Inc., Portsmouth, NH, USA).

The epoxide fraction was quantified by ^1^H NMR (nuclear magnetic resonance) spectroscopy (USA AC-P 400 MHz spectrometer, Bruker, Portsmouth Carteret, NJ, USA) at room temperature. Epoxidized GNR was dissolved in deuterated chloroform. The epoxide fraction was calculated by the ratio of integral peak areas in NMR spectra. The peaks at chemical shifts of 2.7 and 5.1 ppm represent the epoxy proton and alkene proton in epoxidized GNR, respectively [12]. The epoxide fraction was calculated by the equation:

(1) $$Epoxide\;fraction=\frac{A_{2.7}}{A_{2.7}+A_{5.1}}\times 100\%$$

where $A_{2.7}$ and $A_{5.1}$ are integral peak areas at 2.7 and 5.1 ppm chemical shifts in the NMR spectra.

GNR/EGNR blends were compounded with CB, antioxidant and sulfur-based curing packages (Table 1) in a Banbury mixer (Farrel-Birmingham CO, Buffalo, NY, USA) at a fill factor of 0.7. The ratio of GNR/epoxidized GNR blend was constant: 0.7:0.3. Rubber was mixed with fillers, processing aids and antioxidant (N-(1,3-dimethylbutyl)-N’-phenyl-p-phenylenediamine (6PPD)) for 12 min, then stearic acid, zinc oxide, TBBS and sulfur were added and mixed into the rubber compounds for 3 min. Compounding started at 85 °C and 60 rpm. Consumed power (kw) of compounding in the Banbury mixer was recorded at 1 point/second with Pro-server Ex software version 1.3 (Pro-face Digital Electronics CO., Osaka, Japan). Energy consumption was calculated by the average value of power consumption rate from feeding rubber into the Banbury mixer to the end of mixing. After mastication, the rubber compounds were passed nine times through a two-roll mill (roll diameter 15.24 and 33.02 cm width) (Rubber City Machinery Corporation, Akron, OH, USA).

Curing behavior was analyzed using a Monsanto Rheometer ODR 2000, according to ASTM D2084. The curing rate was calculated by the curing rate index [13]:

(2) $$Curing\;rate\;index=\frac{100}{T_{90}-TS_{2}}$$

where *T*_90_ is the time when torque reached 90% of maximum torque, and *TS*_2_ is the time when torque increased two units above minimal torque.

Samples were cured in a heat compression mold with 16 tons of force at 160 °C, according to ASTM D3182. The curing time was T_90_ with an additional 5 min in order to completely vulcanize each compound.

The loss modulus E’’ and storage modulus E’ were measured by the dynamic mechanical analyzer Q800 (TA Instruments, New Castle, UK), using a sample size of 18 mm × 3 mm × 2 mm (length × width × thickness). Testing conditions were 10 Hz from −90 °C to 90 °C, at 3 °C/min, and 10% strain amplitude; Tan δ, which estimates the relative amount of viscous and elastic portions in the composite, was calculated by the ratio of E’’ over E’. Rolling resistance (energy loss during continuous deformation) and wet grip (friction between wet surface and rubber material) were estimated by the tan δ at 60 °C and 0 °C [14,15,16].

The crosslink density and gel fraction were measured by swelling tests, according to ASTM D6814. Rubber composites were immersed in 40 mL toluene at 23 ± 2 °C for 96 h, and toluene was replaced every 24 h. Crosslink density was calculated by the Flory-Rehner equation [17,18]:

(3) $$-\ln\left(1-V_{r}\right)-V_{r}-\chi V_{r}{}^{2}=V_{s}\eta_{\mathrm{swell}}\left(V_{r}^{\frac{1}{3}}-\frac{V_{r}}{2}\right)$$

Since all composite samples were made with the same filler and constant filler loading, crosslink density was compared without Kraus correction [19,20].

Χ is the rubber-solvent interaction parameter. The NR-toluene interaction parameter is 0.391 [21]. The actual EGNR-toluene interaction parameter could not be calculated because it was not possible to measure the osmotic potential of the EGNR solution. Therefore, 0.391 was used as the parameter of both GNR-toluene and EGNR-toluene interactions, but it is likely that EGNR may have poorer interaction and higher χ with toluene than GNR. $\eta_{\mathrm{swell}}$ is the crosslink density of rubber (kmol/m^3^). $V_{s}$, the molar volume of toluene, is 106.27 cm^3^/mol [22]. *V_r_* is the volume fraction of the rubber in swollen gel, which was measured by equation:

(4) $$V_{r}=\frac{V_{rubber}}{V_{solvent}+V_{rubber}}=\left(\frac{m_{d}-m_{b}\times f}{\rho_{rubber}}\right)÷\left[\frac{m_{s}-m_{d}}{\rho_{solvent}}+\left(\frac{m_{d}-m_{b}\times f}{\rho_{rubber}}\right)\right]$$

where $V_{rubber}$ and $V_{solvent}$ are the volume of the rubber matrix and toluene in swollen gel, respectively. $m_{b},\;m_{d},m_{s}$ are sample weights (to an accuracy of 1 mg using a Model ME54E, Mettler analytical balance, Columbus, OH, USA): $m_{b}$ is the initial weight, $m_{s}\;$ is the swollen weight and $m_{d}$ is the weight after the swollen samples were dried. f is the weight fraction of non-rubber components (including filler, curing packages, antioxidant and processing aids). The densities of the rubber matrices (GNR, HNR and SBR) and toluene are $\rho_{rubber}$ and $\rho_{solvent}$, respectively. GNR and EGNR $\rho_{rubber}$ values were 0.92 and 0.96 g/cm^3^, respectively, and $\rho_{solvent}$ was 0.867 g/cm^3^. Gel fraction was calculated from $m_{d}$ divided by $m_{b}$.

Following ASTM D-412, the mechanical properties of cured rubber samples were assessed using test dumbbells cut with a die, size D (CCSi Inc. Akron. OH, USA). Tensile strength, elongation at break and modulus at 100% elongation were quantified by tensiometry (tensiometer Model 3366, Instron, Norwood, MA, USA) at ambient temperature (23 ± 2 °C). An elongation axial extensometer (Model 3800, Epsilon Technology Corp., Jackson, WY, USA) was used to calibrate strain level and elongation at break. Hardness number was quantified according to ASTM D 2240 using a Shore A durometer (Model 408, PTC Instruments, Los Angeles, CA, USA) affixed to an operating type 2 stand (Model 472, PTC Instruments, Los Angeles, CA, USA). All tests were performed in triplicate.

The cured rubber composites were submerged in naphthenic oil at ambient temperature (23 ± 2 °C). Oil resistance was estimated by the weight change during immersion, calculated by the equation:

(5) $$Weight\;change=\frac{m_{2}-m_{1}}{m_{1}}\times 100\%$$

where $m_{1}$ and $m_{2}$ are weights of rubber composites before and after immersion in naphthenic oil.

## 3. Results and Discussion

### 3.1. EGNR Characterization

**Figure 1:**
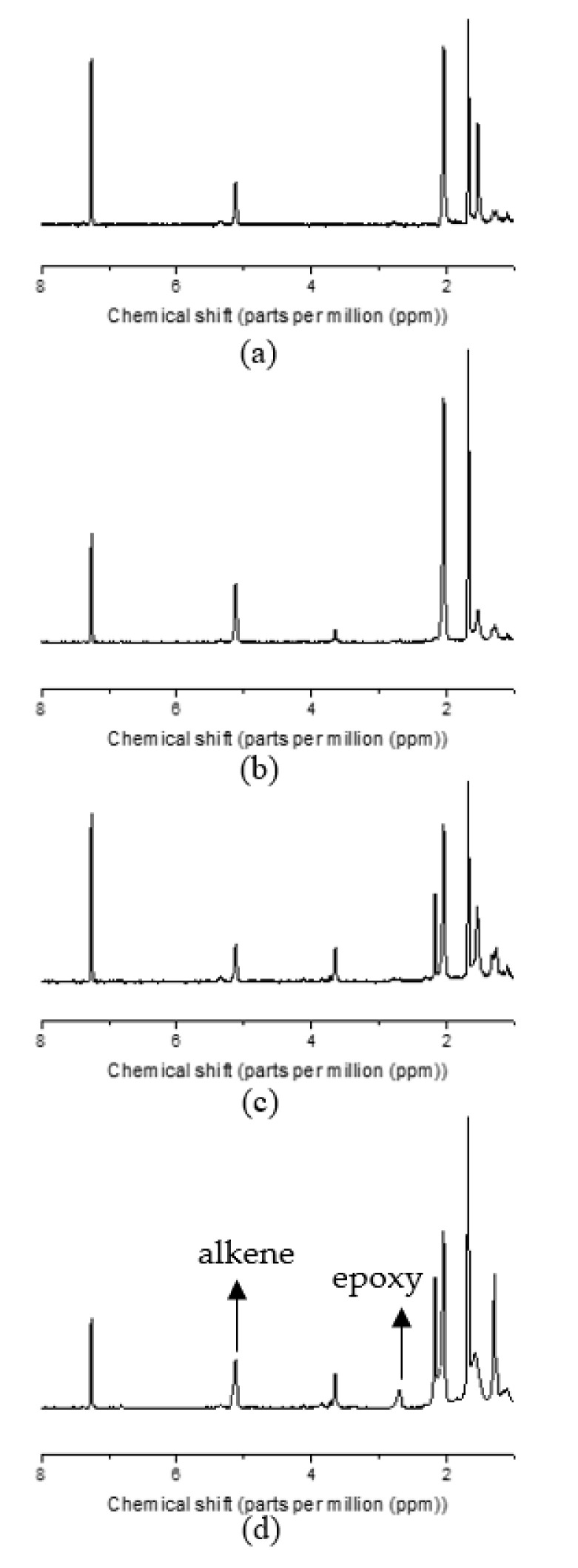
NMR spectra of guayule natural rubber (GNR) and epoxidized guayule natural rubber (EGNR): (**a**) GNR; (**b**) EGNR (mole ratio = 0.15); (**c**) EGNR (mole ratio = 0.30); (**d**) EGNR (mole ratio = 0.45).

**Figure 2:**
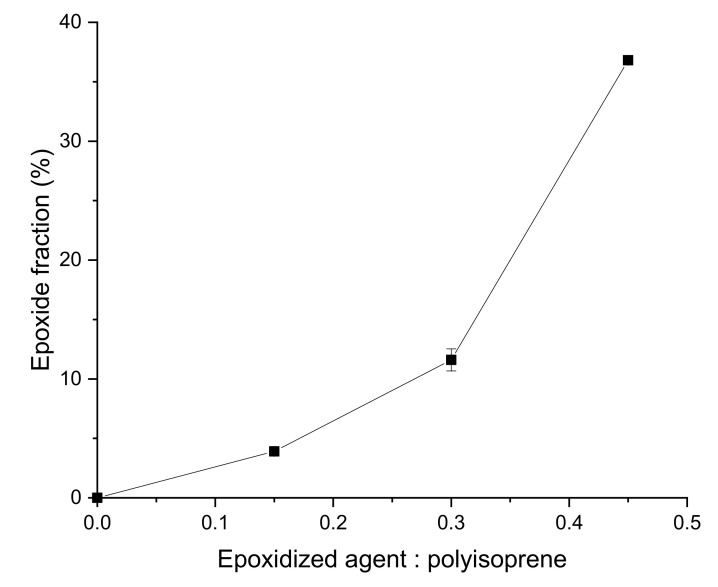
Epoxide fraction of epoxidized guayule natural rubber.

^1^H NMR results confirmed the success of epoxidation. The height of the peak assigned to epoxy protons was enhanced with increasing ratio of formic acid to polyisoprene units, which indicated that more C=C double bonds were converted to epoxide groups (Figure 1). In addition, the epoxide fraction increased as more epoxidized agent was added (Figure 2). The peak at 3.7 ppm, which was seen in all EGNRs was attributed to protons of R-O-CH_2_-R groups in residual, Triton X-100 surfactant [23].

### 3.2. Processability

Epoxidation had little effect on the energy consumption of rubber compounding (Table 2). As the epoxide fraction increased up to 11.6%, the curing curves of composites made with GNR/EGNR blends and GNR alone overlapped before reaching maximal torque. GNR/EGNR blends also have lower T90 and higher curing rate index than GNR composites because epoxide groups can activate the adjacent double bonds.

**Figure 3:**
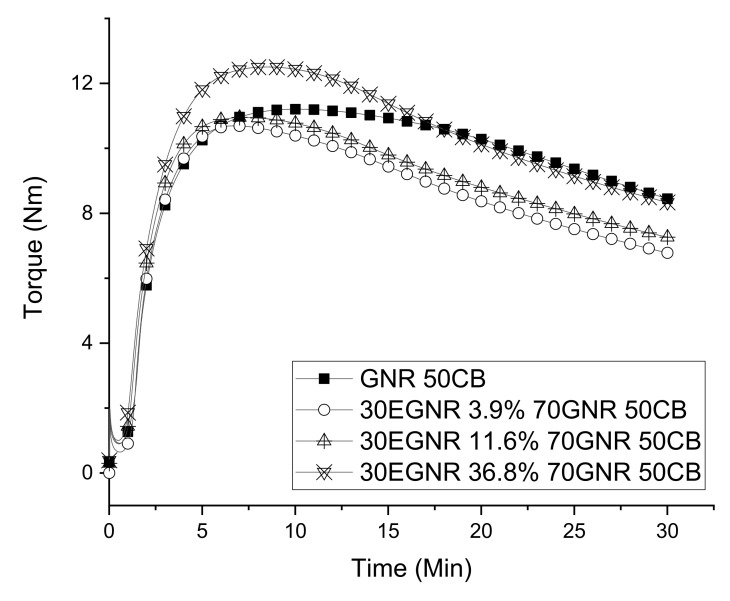
Curing curves of guayule natural rubber/epoxidized guayule natural rubber blend composites with various epoxide fractions. GNR 50CB: guayule natural rubber composites without epoxidation; 30EGNR 3.9% 70GNR 50CB: guayule natural rubber/epoxidized guayule natural rubber blend composites with 3.9% epoxide fraction; 30EGNR 11.6% 70GNR 50CB: guayule natural rubber/epoxidized guayule natural rubber blend composites with 11.6% epoxide fraction; 30EGNR 36.8% 70GNR 50CB: guayule natural rubber/epoxidized guayule natural rubber blend composites with 36.8% epoxide fraction.

The GNR/EGNR blend composite with 36.8% epoxide fraction had higher maximal torque than other composites due to the ring-open reaction between epoxy groups of EGNR and polar–polar interactions formed between rubber epoxide groups (Figure 3 and Table 3). The accelerator used in this study (TBBS) can have a ring-opening reaction with epoxide groups to generate hydroxyl groups, which can react with other epoxide to form additional crosslinks [3].

After reaching maximal torque, GNR/EGNR blend composites had more rapid reversion than GNR composites. Reversion is due to the decomposition of polysulfide crosslinks in cured rubber composites [24]. Epoxidation reduces the unsaturated bonds which act as crosslinking sites for sulfur. Because all the formulations were made using a fixed amount of sulfur (Table 1), the excess available sulfur may cause more polysulfide and less monosulfide crosslinks to be formed during vulcanization [25]. Thus, epoxidation increased the reversion of the rubber composites.

Maximal torque and the difference between maximal and minimal torque (MH-ML) changed little as the epoxide fraction increased from 0 to 11.6%. The similar MH-ML can be related to the balance between reversion and ring-opening reaction. Increasing the epoxide fraction can reduce fraction of unsaturated bonds and enhance both reversion and additional crosslinks formed by ring-opening reaction. The relationship between crosslink density and torque difference can be a topic of future studies.

### 3.3. Crosslink Density and Gel Fraction

**Figure 4:**
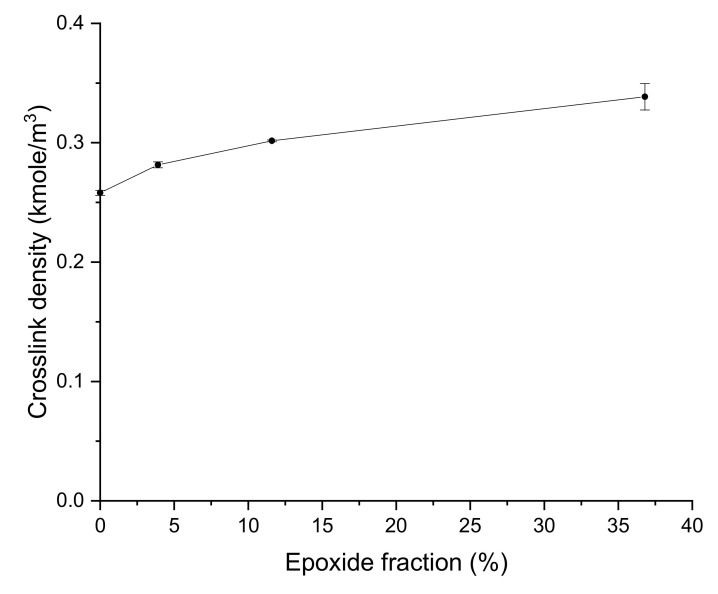
Crosslink density of guayule natural rubber/epoxidized guayule natural rubber blend composites at various epoxide fractions.

**Figure 5:**
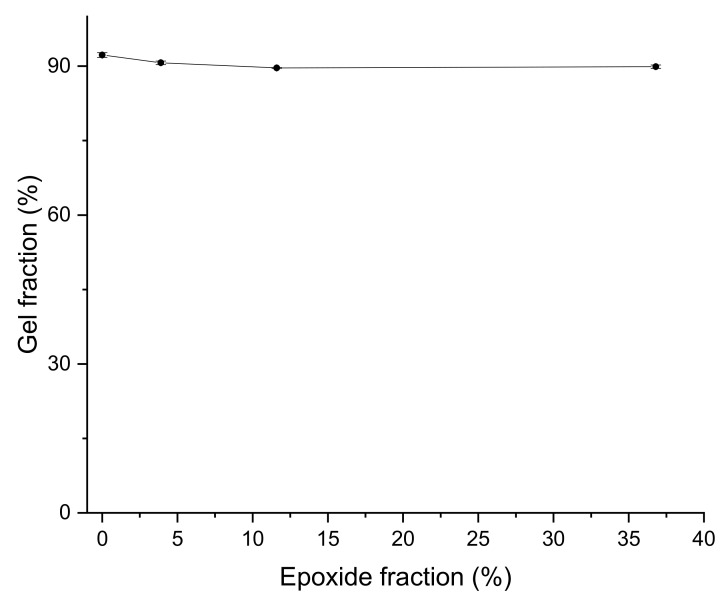
Gel fraction of guayule natural rubber/epoxidized guayule natural rubber blend composites at various epoxide fractions.

The crosslink density of GNR/EGNR blend composites increased as more unsaturated bonds were converted to epoxide groups (Figure 4 and Table 4), which can be explained by the ring-opening reactions between epoxy groups and activated double bonds by the adjacent epoxy groups of EGNR [26]. In addition, the hindrance of the epoxide groups and the polar interactions of epoxidized rubber molecules restrict the swelling behavior of rubber composites. These factors contribute to the increase in crosslink density of GNR/EGNR blend composites with EGNR (Figure 4). Similar effects were also reported in epoxidized SBR composites [10]. The gel fraction was independent of epoxidation (Figure 5 and Table 4) because gel fraction is less sensitive to changing crosslink density at a higher gel fraction region; a similar phenomenon was reported in vulcanized SBR compounds [27].

### 3.4. Mechanical Properties

Tensile strength and elongation at break reduced while modulus at 100% strain and hardness number increased as epoxide fraction increased (Table 5). A similar phenomenon was reported in epoxidized Hevea NR composites [25]. Epoxidation reduced strain-induced crystallization of NR, which reduced elongation at break and tensile strength. The increased modulus and hardness number resulted from reduced chain mobility. Increased glass transition temperature was observed in epoxidized Hevea NR, which indicated free volume of chain [28]. In addition, the increased crosslink density we discussed in the previous section also contributed to reduced elongation at break and increased stiffness of epoxidized GNR composites. Similar effects of epoxidation on SBR was also reported, and EGNR composites in this research had a higher hardness number than the epoxidized SBR [10].

### 3.5. Oil Resistance

**Figure 6:**
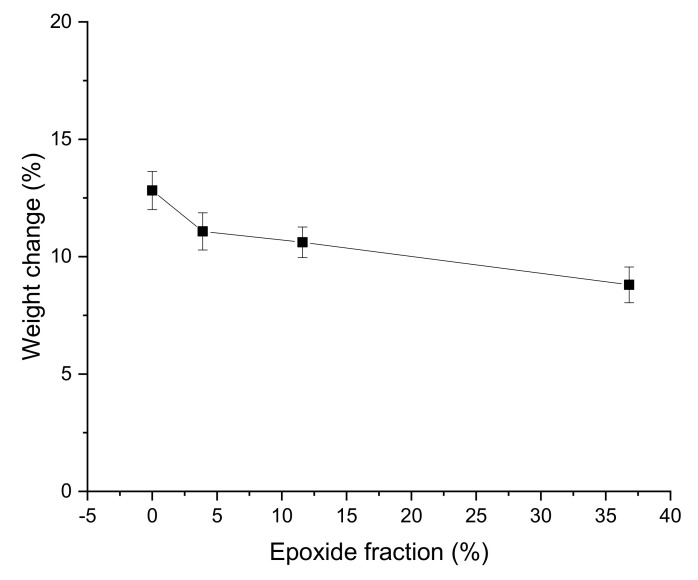
Weight change during immersion in oil of guayule natural rubber/epoxidized guayule natural rubber blend composites at various epoxide fractions.

Weight change after oil immersion decreased with increasing epoxidation level (Figure 6), indicating that the chemical modification improved oil resistance as predicted. EGNR has a higher polarity than GNR, which leads to a weaker interaction with saturated hydrocarbons, such as naphthenic oil [28]. Thus, the swelled EGNR/GNR blend composites absorbed less oil than the GNR composites during oil immersion.

### 3.6. Dynamic Mechanical Properties

**Figure 7:**
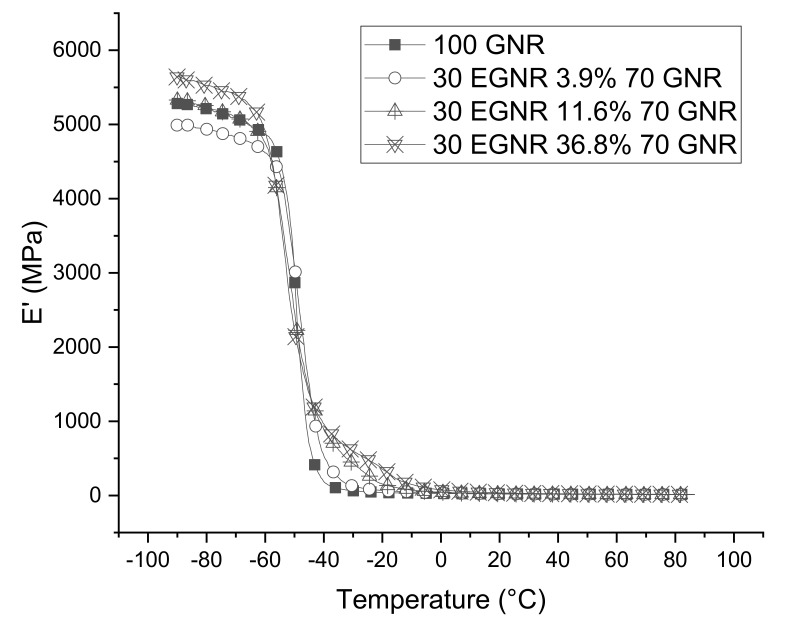
Elastic modulus (E’) of guayule natural rubber/epoxidized guayule natural rubber blend composites with various epoxide fractions. GNR 50CB: guayule natural rubber composites without epoxidation; 30EGNR 3.9% 70GNR 50CB: guayule natural rubber/epoxidized guayule natural rubber blend composites with 3.9% epoxide fraction; 30EGNR 11.6% 70GNR 50CB: guayule natural rubber/epoxidized guayule natural rubber blend composites with 11.6% epoxide fraction; 30EGNR 36.8% 70GNR 50CB: guayule natural rubber/epoxidized guayule natural rubber blend composites with 36.8% epoxide fraction.

**Figure 8:**
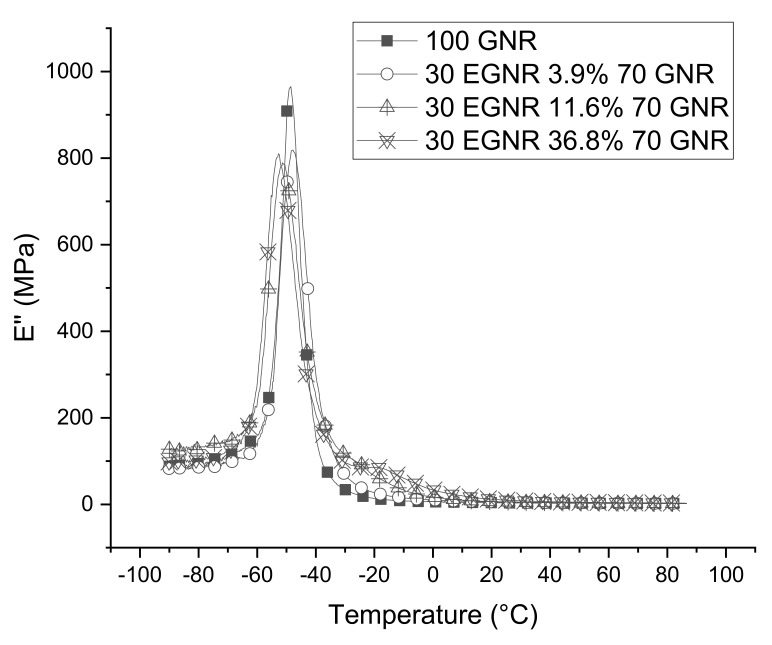
Loss modulus (E’’) of guayule natural rubber/epoxidized guayule natural rubber blend composites with various epoxide fractions. GNR 50CB: guayule natural rubber composites without epoxidation; 30EGNR 3.9% 70GNR 50CB: guayule natural rubber/epoxidized guayule natural rubber blend composites with 3.9% epoxide fraction; 30EGNR 11.6% 70GNR 50CB: guayule natural rubber/epoxidized guayule natural rubber blend composites with 11.6% epoxide fraction; 30EGNR 36.8% 70GNR 50CB: guayule natural rubber/epoxidized guayule natural rubber blend composites with 36.8% epoxide fraction.

E’ and E” of GNR/EGNR blends are higher than GNR because epoxidation increased glass transition temperature (Figure 7 and Figure 8). Epoxidation can increase rotation hindrance of GNR and reduce free volume of chain segments. A similar phenomenon was also reported in epoxidized SBR and Hevea natural rubber composites [10,28].

**Figure 9:**
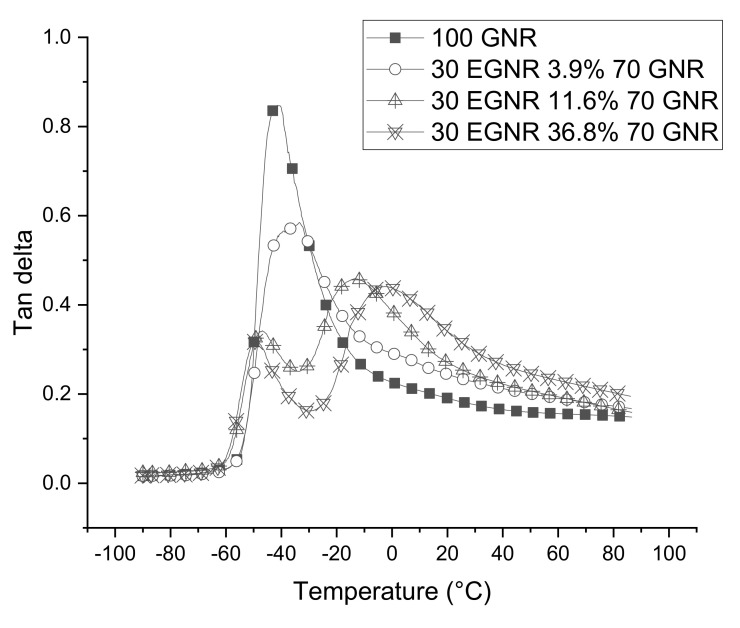
Tan δ of guayule natural rubber/epoxidized guayule natural rubber blend composites with various epoxide fractions. GNR 50CB: guayule natural rubber composites without epoxidation; 30EGNR 3.9% 70GNR 50CB: guayule natural rubber/epoxidized guayule natural rubber blend composites with 3.9% epoxide fraction; 30EGNR 11.6% 70GNR 50CB: guayule natural rubber/epoxidized guayule natural rubber blend composites with 11.6% epoxide fraction; 30EGNR 36.8% 70GNR 50CB: guayule natural rubber/epoxidized guayule natural rubber blend composites with 36.8% epoxide fraction.

Tan δ peaks indicated the glass transition zones of composites made with GNR/EGNR blends or GNR alone (Figure 9). The free volume of GNR molecules drastically increased with rising temperature, as rubber converted from a glassy to rubbery state, resulting in high energy dissipation and a maximal tan δ peak [14]. However, in 3.9% epoxide fraction composites, the tan δ peak height decreased because the epoxide groups restricted the mobility of rubber molecules which, in turn, reduced the energy dissipated at low temperatures. As the epoxide fraction further increased, the glass transition zones of EGNR and GNR separated into different temperature regions, with GNR being the lower temperature peak and EGNR the higher of the two, again due to reduced chain mobility and more epoxy groups [12]. The predicted wet traction almost doubled with epoxidation, while rolling resistance was increased only 1.4 fold (Table 6), suggesting that EGNR may have potential to improve tire safety.

## 4. Conclusions

Epoxidation improved the oil resistance of GNR, increased composite stiffness and increased predicted wet traction more than predicted rolling resistance. These properties may allow new EGNR products to be developed which can be in direct contact with oil, such as sealing parts in combustion engines, and may lead to improved tire safety. This EGNR has similar stiffness to some SRs, including the commonly used SBR, and should increase the number of products in which SR can be replaced by EGNR, improving the sustainability of the rubber industry. In addition, the epoxidation methods developed in this research may replace the current epoxidation process with organic solvent to reduce VOC generation from the rubber production process.

## Figures and Tables

**Table 1:** Rubber compound formulae.

Material	Phr			
GNR	100	70	70	70
EGNR (mole ratio = 0.15)	0	30	0	0
EGNR (mole ratio = 0.3)	0	0	30	0
EGNR (mole ratio = 0.45)	0	0	0	30
Carbon black N330	50	50	50	50
Sulfur	4.5	4.5	4.5	4.5
ZnO	5	5	5	5
TBBS	1	1	1	1
Stearic acid	1	1	1	1
6PPD	2	2	2	2

**Table 2:** Energy consumption of guayule natural rubber/epoxidized guayule natural rubber blend composites at various epoxide fractions.

Epoxide Fraction (%)	Average Energy Consumption, kw
0	20
3.9	19
11.6	19
36.8	21

**Table 3:** *TS*_2_, T90, Curing rate index, minimal torque (ML), maximal torque (MH) and difference between maximal and minimal torque (MH-ML) of guayule natural rubber/epoxidized guayule natural rubber blend composites at various epoxide fractions.

Epoxide Fraction, (%)	*TS*_2_ (min)	T90 (min)	Curing Rate index	ML (NM)	MH(Nm)	MH-ML (Nm)
0	0.93	4.87	25.38	0.92	11.21	10.29
3.9	0.98	4.00	33.11	0.65	10.69	10.05
11.6	0.87	3.78	34.36	0.90	10.96	10.06
36.8	0.79	4.37	27.93	1.00	12.51	11.50

**Table 4:** Volume fraction of the rubber network in the swollen phase, crosslinks density of guayule natural rubber/epoxidized guayule natural rubber blend composites at various epoxide fractions.

Epoxide Fraction, %	Volume Fraction of the Rubber, %	Crosslinks Density, kmol/m^3^
0	25.46	0.26
3.9	26.38	0.28
11.6	27.12	0.30
36.8	28.40	0.34

**Table 5:** Tensile strength, elongation at break, modulus at 100% strain and hardness number of guayule natural rubber/epoxidized guayule natural rubber blend composites at various epoxide fractions.

Epoxide Fraction, (%)	Tensile Strength, Mpa	Standard Deviation	Elongation at Break, %	Standard Deviation	Modulus at 100% Strain, Mpa	Standard Deviation	Hardness Number	Standard Deviation
0	22.9	0.4	301.0	4.3	6.5	0.1	74.2	0.4
3.9	22.0	0.1	273.1	2.2	7.8	0.1	75.4	0.5
11.6	19.8	0.5	219.4	3.8	9.0	0.1	78.2	0.8
36.8	18.8	0.4	197.7	5.4	9.9	0.1	80.2	1.1

**Table 6:** Estimated wet traction and rolling resistance of guayule natural rubber (GNR) and GNR/epoxidized guayule natural rubber (EGNR) blend composites.

Epoxide Fraction (%)	Tan Delta at 0 °C (Wet Traction)	Tan Delta at 60 °C (Rolling Resistance)
0	0.23	0.16
3.9	0.29	0.19
11.6	0.38	0.19
36.8	0.44	0.23

## Data Availability

Not applicable.
